# Long-Chain Fatty Acids Alter Estrogen Receptor Expression in Breast Cancer Cells

**DOI:** 10.3390/ijms26146722

**Published:** 2025-07-13

**Authors:** Ruiko Ogata, Yi Luo, Rina Fujiwara-Tani, Rika Sasaki, Ayaka Ikemoto, Kaho Maehana, Ayaka Sasaki, Takamitsu Sasaki, Kiyomu Fujii, Hitoshi Ohmori, Hiroki Kuniyasu

**Affiliations:** Department of Molecular Pathology, Nara Medical University School of Medicine, Kashihara 634-8521, Japan; pkuma.og824@gmail.com (R.O.); lynantong@hotmail.com (Y.L.); rika0st1113v726296v@icloud.com (R.S.); a.ikemoto.0916@gmail.com (A.I.); dc122091@naramed-u.ac.jp (K.M.); ayaka.med.0523@gmail.com (A.S.); takamitu@fc4.so-net.ne.jp (T.S.); toto1999-dreamtheater2006-sms@nifty.com (K.F.); brahmus73@hotmail.com (H.O.)

**Keywords:** long-chain fatty acid, breast cancer, triple negative breast cancer, estrogen receptor

## Abstract

Long-chain fatty acids (LCFAs) have emerged as important regulators of cancer metabolism, but their impact on hormone receptor expression in breast cancer (BCA) remains poorly understood. In this study, we investigated the effects of five LCFAs—linoleic acid (LA), oleic acid (OA), elaidic acid (EA), palmitic acid (PA), and α-linolenic acid (LNA)—on two BCA cell lines: luminal-type MCF7 and triple-negative MDA-MB-231 (MB231). All LCFAs suppressed cell viability and mitochondrial function in a dose-dependent manner, accompanied by decreased membrane potential, increased reactive oxygen species production, and a metabolic shift. Notably, OA reduced both mRNA and nuclear protein levels of estrogen receptor alpha (ERα) in MCF7 cells, leading to impaired responses to estradiol and tamoxifen. In contrast, PA induced nuclear ERα expression in MB231 cells, although ER signaling remained inactive. MicroRNA profiling revealed that OA upregulated ER-suppressive miR-22 and miR-221 in MCF7, while PA increased miR-34a in MB231, contributing to ERα induction. These findings suggest that specific LCFAs modulate ER expression through epigenetic and post-transcriptional mechanisms, altering hormonal responsiveness in BCA. Our results offer new insights into how dietary lipids may influence therapeutic efficacy and tumor behavior by regulating nuclear receptor signaling.

## 1. Introduction

Breast cancer (BCA) is the most common malignancy among women, with an estimated 2.3 million new cases and 680,000 deaths worldwide in 2020 [1]. Its incidence is higher in high-income countries, while mortality is disproportionately greater in low- and middle-income countries, reflecting regional disparities in early detection and access to treatment, factors that critically impact survival rates.

BCA is classified into four molecular subtypes based on the expression of key biomarkers, each characterized by distinct biological features and therapeutic approaches [2]. The luminal A subtype (estrogen receptor [ER]+/human epidermal growth factor receptor 2 [HER2]−), which expresses hormone receptors, responds well to endocrine therapies, such as tamoxifen and aromatase inhibitors, and is associated with the most favorable prognosis [3]. The luminal B subtype (ER+/HER2+ or high Ki67) typically requires a combination of endocrine therapy with chemotherapy and HER2-targeted agents like trastuzumab [4,5]. The HER2-enriched subtype (ER−/HER2+), once linked to poor outcomes, now shows significantly improved prognoses due to the advent of HER2-targeted therapies including trastuzumab, pertuzumab, and trastuzumab emtansine [6].

In contrast, triple-negative BCA (TNBC; ER−/PR−/HER2−) has limited treatment options and is associated with a poor prognosis. In recent years, however, immune checkpoint inhibitors (e.g., atezolizumab combined with nab-paclitaxel) and poly (ADP-ribose) polymerase inhibitors (e.g., olaparib) have been approved for specific patient subgroups [7,8]. Furthermore, intratumoral heterogeneity and differences in metastatic behavior among subtypes underscore the increasing importance of personalized medicine [9,10,11].

ERα is a nuclear steroid receptor expressed in approximately 70% of BCAs and plays a central role in regulating cell proliferation, differentiation, and tumor progression [12,13]. ERα is activated in an estrogen-dependent manner and promotes tumor cell growth by modulating the transcription of target genes via estrogen response elements (EREs) [14]. The *ER* gene (*ESR1*) contains complex transcriptional regulatory regions and is subject to epigenetic regulation, including histone acetylation and DNA methylation [15]. For instance, CpG methylation of the ESR1 promoter is associated with ER silencing and is frequently observed in triple-negative breast cancer (TNBC) [16]. *ER* expression is maintained by transcription factors such as GATA-binding protein 3 (*GATA3*) and forkhead box protein A1 (FOXA1), which regulate chromatin accessibility at the *ER* locus [17,18]. Notably, GATA3 is essential for maintaining luminal differentiation in BCA and functions alongside ER as a “luminal master regulator” [19]. Several microRNAs (miRNAs) directly target ER mRNA and regulate its expression through translational repression or mRNA degradation. For example, miR-221/222, miR-206, and miR-22 have been shown to bind to the 3′UTR of ESR1, leading to reduced ER protein levels [20,21].

Long-chain fatty acids (LCFAs), defined as fatty acids with 12 or more carbon atoms, are integral to membrane structure, energy metabolism, and cell signaling. Recent studies have highlighted their important roles in cancer cell proliferation, invasion, and metabolic adaptation [22]. In addition to exhibiting the Warburg effect and a dependence on glucose metabolism, cancer cells promote tumor progression by modulating fatty acid uptake, synthesis, and oxidation [23,24]. Elevated expression of CD36 and fatty acid transport proteins (FATPs) enhances LCFA uptake, which is subsequently used for mitochondrial β-oxidation or membrane lipid biosynthesis [25,26,27]. Each LCFA exerts a distinct effect on cancer cells. Palmitic acid (PA, C16:0) contributes to cancer cell inflammation and increases the metastatic potential by inducing endoplasmic reticulum stress, altering lipid rafts, and activating toll-like receptor 4 [28]. Oleic acid (OA, C18:1) promotes cell proliferation in certain cancers (e.g., breast and prostate cancers) but also facilitates drug resistance by suppressing *ER* expression and activating the phosphatidylinositol 3-kinase (PI3K)/AKT pathway [29,30]. Omega-6 fatty acids, such as linoleic acid (LA, C18:2), are considered to promote tumors through the production of inflammatory eicosanoids (e.g., prostaglandin E2) [31,32]. We have previously shown that LA induces dormancy in colorectal cancer cells via the upregulation of miR-497, leading to the suppression of energy metabolism and increased expression of the repressive form of the Gli family zinc finger 2 protein [33,34,35]. This mechanism has attracted attention as a potential cause of delayed metastasis in breast and gastrointestinal cancers. Moreover, LA has been implicated in maintaining stemness in mesenchymal stem cells within the tumor stroma [36], as well as in promoting drug resistance [37] and tumorigenesis [38]. LCFAs have garnered increasing attention as tumor-promoting factors in various cancers. In TNBC, fatty acid oxidation serves as a major source of ATP, and inhibition of the LCFA-metabolizing enzyme carnitine palmitoyltransferase 1A has been proposed as a potential therapeutic strategy [39].

Although LCFAs have been shown to exert various effects on BCA cells, their precise mechanisms, particularly their relationship with hormone receptor expression, remain incompletely understood. In this study, we investigated the potential therapeutic relevance of selected LCFAs by examining their effects on human BCA cell lines.

## 2. Results

### 2.1. Effect of LCFAs on Cell Viability in BCA Cells

**Figure 1:**
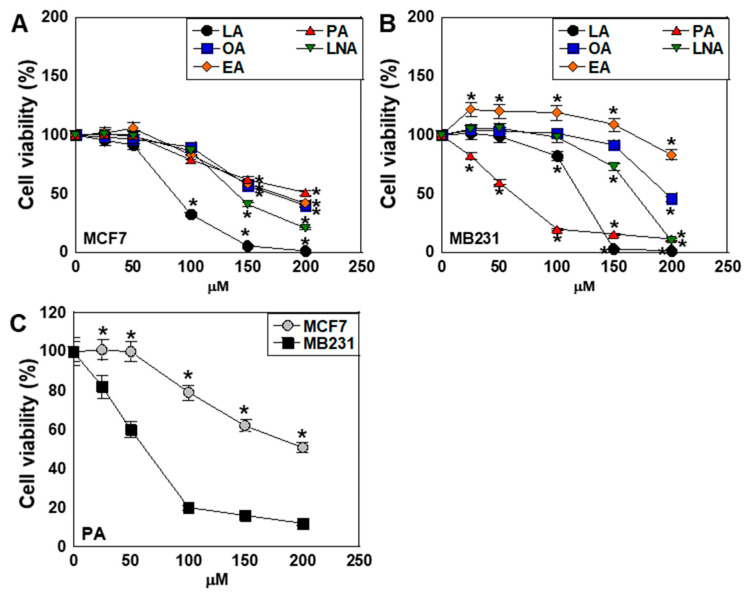
Effect of LCFAs on cell viability of BCA cells. (**A**,**B**) The BCA cell lines MCF7 (**A**) and MT231 (**B**) were treated with various concentrations of LCFAs for 48 h. (**C**) Comparison of PA on cell viability between MCF7 and MB231 cells. Cell viability in untreated controls was set at 100%. Error bars: SD from three independent trials. Statistical analysis: ANOVA with Bonferroni correction. * *p* < 0.05, MCF7 vs. MB231. LCFA, long-chain fatty acid; BCA, breast cancer; LA, linoleic acid; OA, oleic acid; EA, elaidic acid; PA, palmitic acid; LNA, linolenic acid; C, control; ANOVA, analysis of variance; SD, standard deviation.

We first treated MCF7 (estrogen receptor-positive, ER+) and MDA-MB-231 (MB231) (triple-negative breast cancer, TNBC) cells with five types of LCFAs—LA, OA, elaidic acid (EA, C18:1, trans), PA, and α-linolenic acid (LNA, C18:3, ω-3)—at various concentrations. In MCF7 cells, all LCFAs showed dose-dependent viability inhibition, with LA exerting the strongest suppressive effect (Figure 1A). In contrast, MB231 cells were less sensitive to most LCFAs, showing viability inhibition only at higher concentrations, with the exception of PA. Notably, EA promoted cell viability as well. PA, however, exhibited a strong inhibitory effect on MB231 cells, even at low concentrations (Figure 1B). Moreover, a comparison of PA sensitivity revealed that the IC_50_ in MB231 cells was approximately half of that observed in MCF7 cells, indicating a greater inhibitory effect in MB231 (Figure 1C). The treatment concentrations of LCFAs were set to 50 μM based on the results in Figure 1.

### 2.2. Effect of LCFAs on Mitochondrial Function in BCA Cells

**Figure 2:**
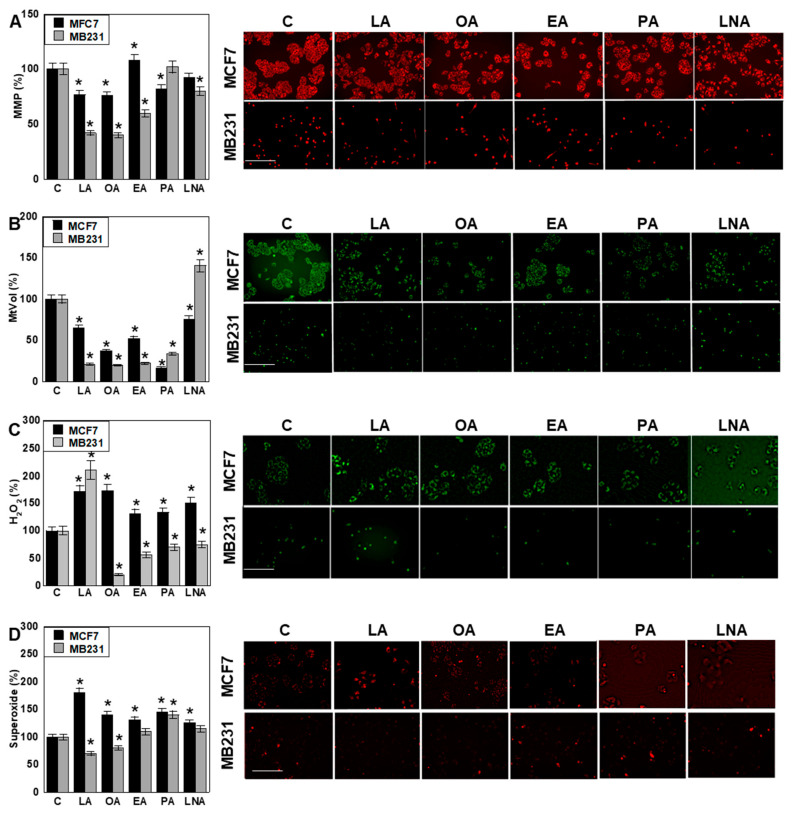
Effect of LCFAs on mitochondrial function in BCA cells. BCA cells were treated with LCFAs (50 μM) for 48 h. (**A**) MMP, (**B**) MtVol, (**C**) mitochondrial H_2_O_2_, and (**D**) mitochondrial superoxide. Each parameter was expressed relative to the fluorescence intensity of the control, taken as 100%. Scale bar, 50 μm. Error bars: SD from three independent trials. Statistical analysis: ANOVA with Bonferroni correction. * *p* < 0.05 vs. C. LCFA, long-chain fatty acid; BCA, breast cancer; LA, linoleic acid; OA, oleic acid; EA, elaidic acid; PA, palmitic acid; LNA, linolenic acid; C, control; MMP, mitochondrial membrane potential; MtVol, mitochondrial volume; ANOVA, analysis of variance; SD, standard deviation.

As LCFAs are transported into mitochondria via the carnitine shuttle and influence mitochondrial activity [40,41], we next examined their effects on mitochondrial function. In MCF7 cells, mitochondrial membrane potential (MMP) was reduced by LA, OA, and PA, while in MB231 cells, MMP was decreased by LA, OA, EA, and LNA (Figure 2A). Mitochondrial volume (MtVol) decreased with all LCFAs in MCF7 cells. In MB231 cells, MtVol was also reduced by all LCFAs except LNA, which caused an increase in volume (Figure 2B). Mitochondrial H_2_O_2_ levels increased in MCF7 cells with all LCFAs, whereas in MB231 cells, H_2_O_2_ levels rose only with LA and declined with the other fatty acids (Figure 2C). Similarly, mitochondrial superoxide levels increased across all treatments in MCF7 cells, but in MB231 cells, the levels decreased with LA and OA and increased with PA (Figure 2D).

### 2.3. Effect of LCFAs on Stemness and Differentiation of BCA Cells

**Figure 3:**
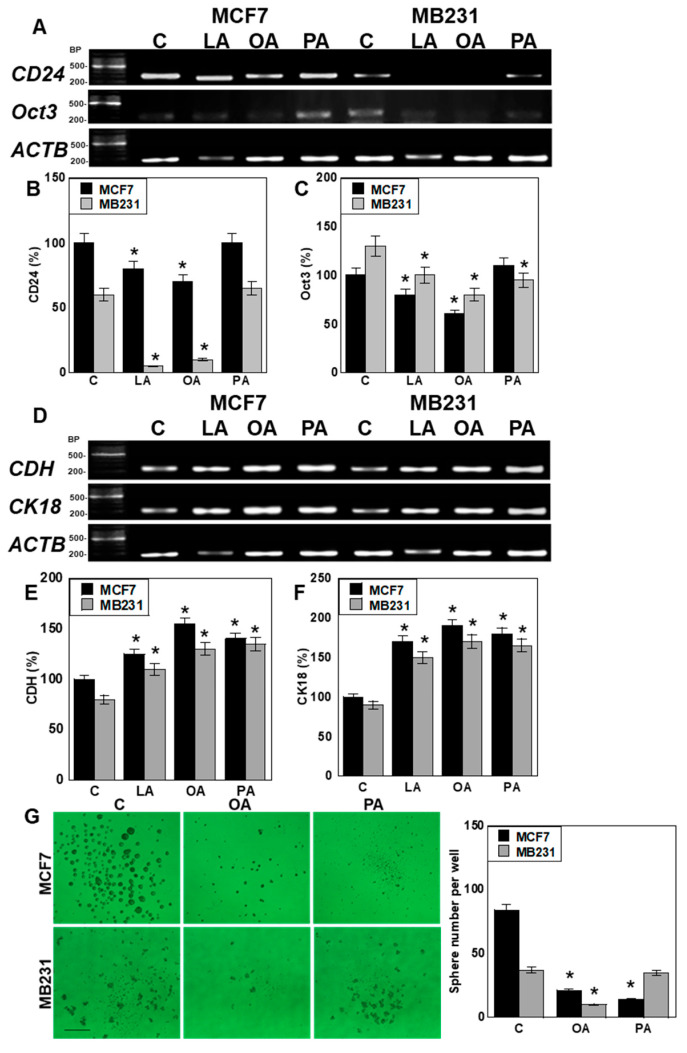
Effect of LCFAs on stemness and differentiation of BCA cells. BCA cells were treated with LCFAs (50 μM) for 48 h. (**A**) Gene expression of stemness-related genes, namely *CD24* and *Oct3*. (**B**,**C**) Semi-quantification of panel A. The MCF7 control was set to 100. (**D**) Expression of differentiation-related genes, namely *CDH* and *CK18*. (**E**,**F**) Semi-quantification of panel D. The MCF7 control was set to 100. (**G**) Sphere formation in the BCA cells. Scale bar, 200 μm. **Right panel**: Quantification of the **left panel**. Error bars: SD from three independent trials. Statistical analysis: ANOVA with Bonferroni correction. * *p* < 0.05 vs. C. LCFA, long-chain fatty acid; BCA, breast cancer; LA, linoleic acid; OA, oleic acid; PA, palmitic acid; C, control; BP, base pair; Oct3, octamer-binding transcription factor 3; ACTB, β-actin; CDH, E-cadherin; CK18, cytokeratin; ANOVA, analysis of variance; SD, standard deviation.

We next investigated the effects of LCFAs on stemness by analyzing the expression of *CD24* and octamer-binding transcription factor 3 (*Oct3*), which are recognized markers of stemness in BCA [42]. *CD24* expression was reduced by LA and OA in both the MCF7 and MB231 cell lines (Figure 3A,B). *Oct3* expression was decreased by LA and OA in MCF7 cells, whereas in MB231 cells, all LCFAs led to reduced *Oct3* expression (Figure 3A,C). To assess differentiation, we examined the expression of the epithelial markers E-cadherin *(CDH*) and cytokeratin 18 (*CK18*). Both genes were upregulated by all LCFAs in both cell lines (Figure 3D–F). In sphere formation, OA reduced the sphere number in MCF7 and MB231 cells. PA reduced the sphere number in MCF7 cells alone (Figure 3G).

### 2.4. Effect of LCFAs on Energy Metabolism of BCA Cells

**Figure 4:**
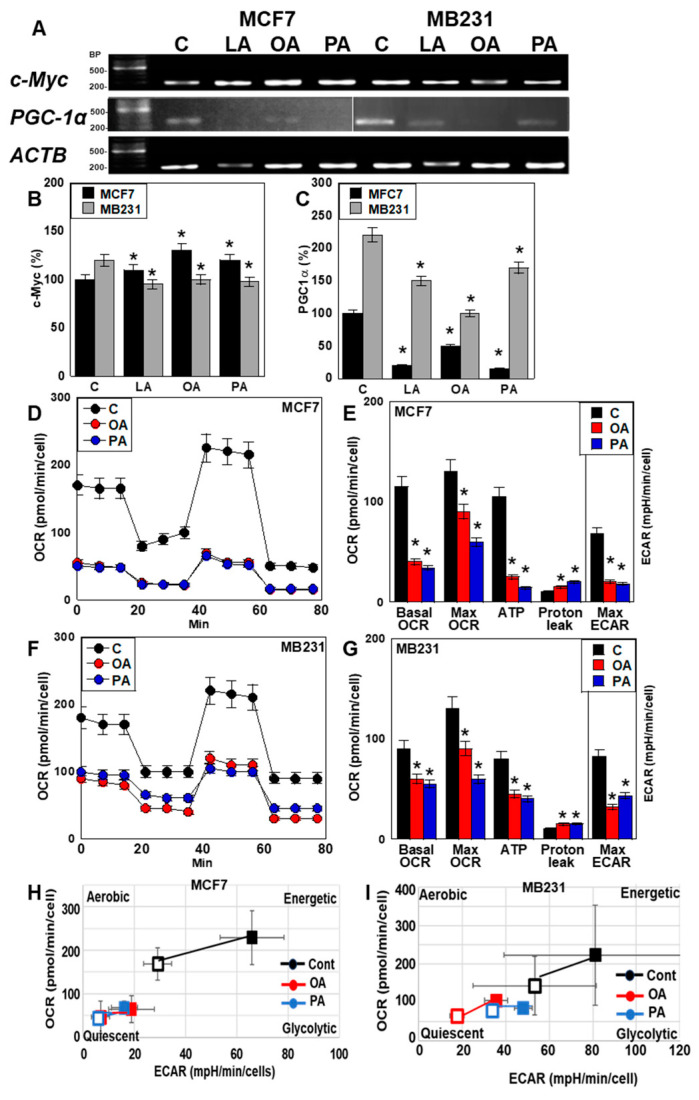
Effect of LCFAs on energy metabolism of BCA cells. BCA cells were treated with LCFAs (50 μM) for 48 h. (**A**) Gene expression of energy metabolism-related genes, namely c-Myc and PGC-1α. (**B**,**C**) Semi-quantification of panels (**A**,**D**–**G**). Energy flux analysis of MCF7 (**D**,**F**) and MB231 cells (**E**,**G**). (**H**,**I**) Matrix diagram of energy metabolism. Error bars: SD from three independent trials. Statistical analysis: ANOVA with Bonferroni correction. * *p* < 0.05 vs. C. LCFA, long-chain fatty acid; BCA, breast cancer; LA, linoleic acid; OA, oleic acid; PA, palmitic acid; C, control; BP, base pair; PGC-1α, peroxisome proliferator-activated receptor gamma coactivator 1-α; ACTB, β-actin; OCR, oxygen consumption rate; ECAR, extracellular acidification rate; Max maximal; C, control; cont, control; ANOVA, analysis of variance; SD, standard deviation.

To assess the impact of LCFAs on energy metabolism, we analyzed the expression of peroxisome proliferator-activated receptor gamma coactivator-1α (*PGC1α*) (oxidative phosphorylation) and *c-Myc* (glycolysis). In MCF7 cells, *c-Myc* expression was increased by all the LCFAs, whereas in MB231 cells, it was decreased by them (Figure 4A,B). On the other hand, *PGC1α* expression was suppressed by all LCFAs in both MCF7 and MB231 cells (Figure 4A,C). Subsequent flux analysis showed that the basal oxygen consumption rate (OCR), maximal OCR, and ATP production were reduced by all fatty acids in both cell lines. In contrast, the maximal extracellular acidification rate (ECAR) increased in MCF7 cells but decreased in MB231 cells (Figure 4D–G). These results indicated that LCFAs shift energy metabolism toward a glycolytic phenotype in MCF7 cells, whereas they induce a quiescent state in MB231 cells (Figure 4H,I).

### 2.5. Effect of LCFAs on Expression of Estrogen Receptor in BCA Cells

In the luminal-type BCA cell line MCF7, the ER protein was expressed at low levels in the cytoplasm and high levels in the nucleus. Interestingly, treatment with OA led to the disappearance of the nuclear ER protein. In contrast, PA increased ER protein levels in both the cytoplasm and nucleus.

**Figure 5:**
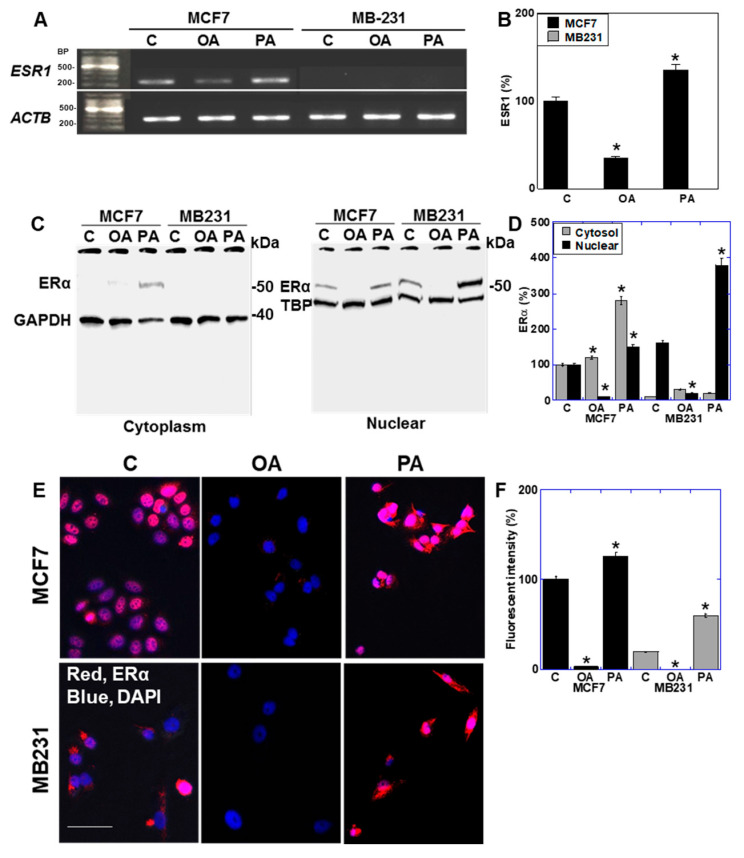
Effect of LCFAs on expression of estrogen receptor in BCA cells. BCA cells were treated with LCFAs (50 μM) for 48 h. (**A**) Gene expression of *ESR1*. (**B**) Semi-quantification of panel A. (**C**) Protein levels of ERα in the cytoplasm and nuclei. (**D**) Semi-quantification of panel D. (**E**) Immunocytochemistry of ERα. Scale bar, 50 μm. (**F**) Semi-quantification of the fluorescence intensity in panel E. Error bars: SD from three independent trials. Statistical analysis: ANOVA with Bonferroni correction. * *p* < 0.05 vs. C.LCFA, long-chain fatty acid; BCA, breast cancer; OA, oleic acid; PA, palmitic acid; C, control; BP, base pair; ESR1, estrogen receptor gene; ER, estrogen receptor; GAPDH, glyceraldehyde-3-phosphate dehydrogenase; TBP, TATA-binding protein; DAPI, 4′,6-diamidino-2-phenylindole; ANOVA, analysis of variance; SD, standard deviation.

In MCF7 cells, OA reduced *ESR1* gene expression. In contrast, in the basal-like TNBC cell line, MB231 showed undetectable expression of ESR in the control and in cells treated with OA or PA (Figure 5A,B). In MB231, ER protein expression was minimal in the cytoplasm; however, nuclear expression was observed, even under control conditions. This nuclear expression was almost completely lost with OA treatment but markedly increased with PA treatment (Figure 5C,D). Fluorescent immunostaining confirmed that nuclear ER expression, as indicated by the co-localization of ER staining with DAPI-stained nuclei, was nearly absent in OA cells and increased with PA in both cell lines (Figure 5E,F). These findings clearly demonstrated that nuclear ER protein levels decreased with OA and increased with PA.

### 2.6. Function of ER Expressed in BCA Cells

**Figure 6:**
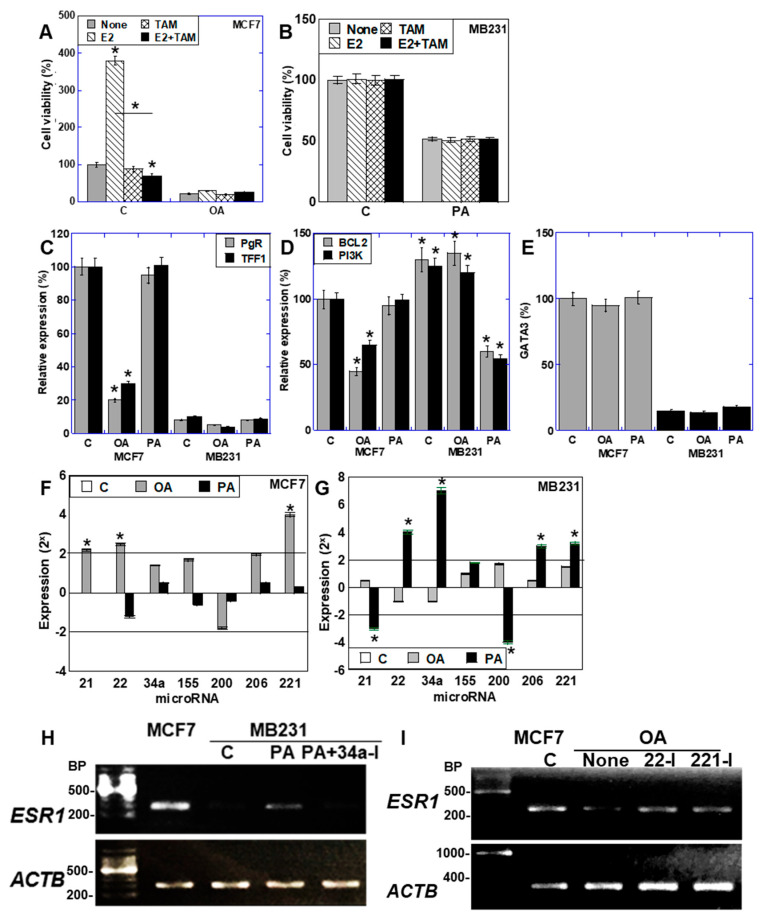
Function of ER expressed in BCA cells. BCA cells were treated with LCFAs (50 μM) with or without E2 or TAM for 48 h. (**A**,**B**) Effects of E2 and TAM on the viability of MCF7 (**A**) and MB231 cells (**B**). (**C**–**E**) Expression of PgR and TFF1 (**C**), BCL2 and PI3K (**D**), and GATA3 (**E**) based on the data from quantitative PCR. The expression was standardized by *ACTB* expression. (**F**,**G**) Expression of ER-associated miRNAs in MCF7 (**F**) and MB231 cells (**G**). (**H**) *ESR1* expression in MB231 cells treated with PA (50 μM) and/or the miR-34a inhibitor (34a-I, 2 μM) for 48 h. (**I**) *ESR1* expression in MCF7 cells treated with OA (50 μM) and/or miR-22 or -221 inhibitors (22-I or 221-I, 2 μM) for 48 h. Error bars: SD from three independent trials. Statistical analysis: ANOVA with Bonferroni correction. * *p* < 0.05 vs. C. LCFA, long-chain fatty acid; BCA, breast cancer; OA, oleic acid; PA, palmitic acid; C, control; ER, estrogen receptor; E2, estradiol; TAM, tamoxifen; PgR, progesterone receptor; TFF1, trefoil factor-1; BCL2, B-cell CLL/lymphoma 2; PI3K, phosphatidyl inositol-3 kinase; GATA3, GATA-binding protein 3; BP, base pair; ANOVA, analysis of variance; SD, standard deviation.

Next, we investigated the function of ER expression in BCA cells. First, we examined responses to estradiol (E2) and tamoxifen (TAM) (Figure 6A,B). In MCF7 cells, E2 enhanced cell viability under control conditions; however, this effect was abolished by co-treatment with TAM (Figure 6A). When treated with OA, MCF7 cell viability was reduced in comparison with the untreated control. Moreover, compared with the untreated control, OA did not significantly alter the effects of E2, TAM, or E2 + TAM, suggesting that suppression of ER expression by OA reduced responsiveness to E2 and TAM. In contrast, MB231 cells showed no response to either E2 or TAM under control conditions. Although PA induced ER expression in MB231 cells, these cells exhibited a reduced or no response to E2 or TAM (Figure 6B).

Next, we examined the expression of ER target genes such as progesterone receptor (*PgR*) and trefoil factor 1 (*TFF1*). In MCF7 cells, the expression of both genes was decreased by OA treatment, but it was unchanged by PA. In MB231 cells, the expression levels of both genes remained low with no significant changes (Figure 6C). We also assessed the expression of the ER target gene *B-cell/CLL lymphoma 2* (*BCL2*), which promotes BCA cell survival, and *PI3K*, an ER-interacting factor. In MCF7 cells, both genes were down-regulated by OA, whereas no changes were observed in MB231 cells. Conversely, PA did not affect their expression in MCF7 cells but significantly reduced their expression in MB231 cells (Figure 6D). The expression of the master regulator of mammary differentiation, GATA3, remained unchanged by both OA and PA in both cell lines (Figure 6E). These findings suggest that, in MCF7 cells, OA suppresses ER expression along with its downstream targets, whereas in MB231 cells, although ER expression is induced by PA, its target genes remain unchanged, indicating that ER is functionally inactive.

We further hypothesized that these changes in gene expression may be mediated by miRNAs and analyzed the expression of miRNAs known to affect the ER [43,44,45,46,47,48,49,50]. In MCF7 cells, OA treatment increased the levels of miR-22 and miR-221, both of which suppressed ER expression. The expression of the anti-apoptotic miRNA miR-21 was also upregulated (Figure 6F). In MB231, OA induced minimal changes in microRNA expression. In contrast, PA treatment increased the levels of ER-suppressive miRNAs miR-22, miR-206, and miR-221 (ER-suppressive miRNAs), whereas miR-200 was downregulated (Figure 6G). Notably, miR-34a, which promotes ER expression, was markedly upregulated, and miR-21 was downregulated. In MCF7, PA had little effect on microRNA expression. To clarify the role of miR-34a in PA-induced *ER* expression in MB231 cells, we examined the effects of an miR-34a inhibitor. miR-34a inhibition suppressed the PA-induced *ERα* expression (Figure 6H). Additionally, inhibition of miR-22 and miR-221 alleviated the OA-induced suppression of *ER* expression in MCF7 cells (Figure 6I).

These findings suggest that coordinated changes in microRNAs contribute to OA-induced ER suppression in MCF7 cells and PA-induced ER expression and apoptosis enhancement in MB231 cells.

## 3. Discussion

In this study, we investigated the effects of five types of LCFAs on BCA cell lines and found that all LCFAs induced dose-dependent inhibition of cell proliferation in both MCF7 and MB231 cells. In BCA, lipid metabolism has been shown to have a strong influence on its malignancy and prognosis [51]. The investigation of the effects of fatty acids on BCA, especially TNBC, is relevant. Notably, OA and PA suppressed mitochondrial function, increased reactive oxygen species (ROS) production, and inhibited energy metabolism, resulting in a shift toward a quiescent phenotype (Table 1). Additionally, suppression of stemness and the epithelial–mesenchymal transition (EMT) was observed. Interestingly, PA induced nuclear ERα expression in MB231 cells—a TNBC cell line—although the receptor appeared to be non-functional. In contrast, OA reduced ERα expression in MCF7 cells, representative of the luminal subtype.

One of the most intriguing findings of this study was the induction of ERα expression by PA in TNBC cells. In luminal-type BCA, ERα is a central driver of tumor proliferation. Previous studies have suggested that PA may influence ERα expression through the induction of cellular stress, including endoplasmic reticulum stress [52,53,54], as well as through epigenetic modifications [55]. In our experiments, PA upregulated ERα protein expression but not mRNA and promoted nuclear translocation of the ERα protein in MB231 cells, a TNBC model. This discrepancy between mRNA and protein may reflect the truncated form of the ERα protein. Although Δ7 ERα has a molecular weight similar to that of normal ERα, it may not be detectable by PCR primers for normal ERα [56]. Δ7 ERα does not exhibit normal receptor activity [57]. Alternatively, even if the ER RNA expression level was undetectable, ER protein accumulation may have occurred because of degradation of ER proteins, which were reduced by inhibition of ubiquitination [58] or enhanced phosphorylation [59]. Although TNBC typically lacks ERα protein expression, the gene itself remains intact; our findings support the notion that its re-expression can be triggered by specific external stimuli [13,60,61]. While the presence or absence of “functionality” is judged solely based on changes in the expression of ER target genes, the direct measurement of transcriptional activity, the confirmation of ER-DNA binding by chromatin immunoprecipitation, and a comprehensive analysis of ER-dependent transcriptional networks have not been performed, so the determination of whether the ER is truly non-functional is limited. Moreover, changes in ERα mRNA and protein expression due to OA and PA have been reported, but direct epigenetic analysis of DNA methylation, histone modification, chromatin accessibility, etc., has not been performed. Although hypotheses have been presented regarding post-translational regulation by endoplasmic reticulum stress and ubiquitination, no experimental data have been presented.

We focused on miRNAs as the potential mechanism underlying this effect. Several microRNAs, including miR-22, miR-206, and miR-221/222, have been reported to suppress ER expression [43,44,45,46,47,48,49], and PA has been shown to further promote the expression of miR-22 and miR-206 [62,63]. In our study, we also observed the upregulation of miR-22, miR-206, and miR-221 following PA treatment. However, PA suppressed miR-200 expression. Reports have demonstrated that PA enhances miR-221/222 expression in hepatocytes and retinal pigment epithelial cells [64,65], suggesting that these microRNAs may indirectly contribute to ERα suppression in TNBC.

In contrast, the expression of miR-34a—known to promote ERα expression [50]—was elevated beyond that of the aforementioned microRNAs. PA-induced upregulation of miR-34a has been previously reported in pancreatic β-cells and hepatocellular carcinoma cells [66,67], suggesting that a similar regulatory response may occur in MB231 cells. However, miR-34a is also known to promote stemness and EMT in TNBC [68]. In our study, treatment with a miR-34a inhibitor suppressed PA-induced ERα expression, supporting the role of miR-34a in PA-mediated upregulation of ERα. These findings suggest that, in this context, the opposing effects of various microRNAs were balanced in a manner that ultimately favored ERα induction. Furthermore, PA may promote chromatin reopening at the *ESR1* locus and enhance gene expression through the activation of peroxisome proliferator-activated receptor (PPAR) γ, accompanied by histone acetylation and recruitment of coactivators such as steroid receptor coactivator-1 (SRC-1) and the cAMP response element-binding protein (CREB)-binding protein (CBP) [69,70]. In addition, PA has been reported to facilitate nuclear translocation of the ERα protein via the activation of the extracellular signal-regulated kinase (ERK) and PI3K/AKT pathways [71]. The lncRNA LYPLAL1-DT suppresses the β-catenin/WNT system and acts as a tumor suppressor, and this lncRNA is induced by FOXO1 [72]. Interestingly, PA suppresses FOXO1 expression [73], which suggests that PA promotes the malignancy of TNBC. However, these mechanisms warrant further investigation in future studies.

One finding of potentially greater clinical significance was the OA-induced loss of ER expression in MCF7 cells. LCFAs such as OA have been reported to reduce ERα mRNA and protein levels, suggesting that the metabolic environment may reprogram hormone sensitivity through epigenetic modifications [74,75]. One proposed mechanism is that OA suppresses protein translation via the endoplasmic reticulum stress response, potentially inhibiting ERα protein synthesis [76,77]. Additionally, OA has been shown to promote ubiquitination and proteasomal degradation of ERα, leading to decreased protein stability [78,79].

Consistent with these mechanisms, our data showed that OA treatment led to a reduction in both ERα mRNA and protein levels. Based on this observation, we examined the involvement of microRNAs and found that OA upregulated the expression of miR-22 [43,80,81,82] and miR-221 [80,82], both known to suppress ERα expression. Moreover, the OA-induced downregulation of ERα was alleviated by treatment with inhibitors of miR-22 and miR-221, supporting their role in mediating OA-driven ER suppression. At the transcriptional level, OA has also been suggested to suppress *ESR1* expression through the activation of peroxisome proliferator-activated receptors (PPARs), particularly PPARα and PPARγ [83].

In this study, we examined the effects of five long-chain fatty acids (LCFAs) on breast cancer (BCA) cells. These LCFAs represent structurally diverse categories, including ω-6, ω-3, saturated, and trans fatty acids. Despite their structural differences, all LCFAs induced mitochondrial dysfunction to varying degrees, as evidenced by decreased mitochondrial membrane potential, reduced mitochondrial mass, increased mitochondrial ROS, and the suppression of energy metabolism. These mitochondrial impairments appear to drive a shift toward a quiescent phenotype, thereby contributing to the suppression of cell proliferation. LA and OA, in particular, led to reduced stemness and enhanced cellular differentiation. Notably, the transition to a quiescent state has been reported to confer chemoresistance in pancreatic cancer cells [84,85]. On the other hand, the suppression of stemness and the promotion of differentiation may instead contribute to reduced metastatic potential and resistance to anticancer drugs [86,87]. Interestingly, the triple-negative breast cancer cell line MB231 exhibited greater resistance to the viability–inhibitory effects of LCFAs compared to luminal-type MCF7 cells, and EA even showed a slight viability-promoting effect in MB231. These findings suggest that LCFAs exert complex and potentially bidirectional effects on BCA cells, with both suppressive and promotive actions depending on the cellular context. Because of their hydrophobic nature, LCFAs can also be readily inserted into the inner mitochondrial membrane, altering membrane fluidity and increasing proton leakage via changes in membrane properties [88]. Consistent with this, our data also demonstrated an increase in proton leakage. LCFA-induced cardiolipin oxidation disrupts respiratory chain function and promotes apoptosis [89]. In addition, LCFAs may enhance mitochondrial ROS production, impair oxidative protein folding in the endoplasmic reticulum, and trigger ER stress.

Our findings suggest that fatty acids modulate the expression profile of BCA cells. Hormone therapy is a key standard treatment for luminal-type BCAs [90], which fundamentally depend on the sustained expression of ER in BCA cells. Our results suggest that OA may suppress *ER* expression, potentially reducing the responsiveness to hormone therapy. OA is a major component of olive oil, comprising over 70% of its content, and is a central component of the Mediterranean diet. OA has been shown to exert beneficial effects, such as anti-inflammatory actions, through various mechanisms, including the activation of sirtuin 1 and PPARα via its metabolite oleoylethanolamide [91]. While the Mediterranean diet has demonstrated a preventive effect against BCA incidence [92], its role in reducing recurrence remains unclear [93]. Further research is needed to clarify the relationship between dietary OA intake and the efficacy of hormone therapy in BCA treatment.

On the other hand, the induction of ER expression by PA in TNBC suggests a potential “luminalization” of TNBC, which could broaden therapeutic strategies. However, in our study, the ER induced in MB231 cells was non-functional. ER dysfunction in TNBC is not solely due to the loss of ER expression but may also result from defects in downstream signaling pathways and the suppression of GATA3, a key regulator of luminal differentiation. ER exerts transcriptional activity through complex formation with coactivators such as SRC-1, SRC-3, and CBP/p300; however, in TNBC, the expression of these coactivators is significantly reduced, making it difficult to restore functional signaling even when ER is re-expressed [94]. Furthermore, ER target genes are often epigenetically repressed in TNBC, further impairing signal transduction [95]. GATA3 plays a critical role in maintaining the luminal lineage of mammary epithelial cells and luminal-type BCA [96,97]. In TNBC—especially in the basal-like subtype—GATA3 expression is frequently diminished or lost, contributing to a breakdown of the luminal phenotype and the acquisition of undifferentiated, mesenchymal-like features [98,99,100]. In our study, PA did not induce GATA3 expression in MB231 cells. Although forced GATA3 expression has been reported to reduce the malignant phenotype of TNBC, it does not result in the full acquisition of a luminal phenotype [101]. These findings underscore that substantial barriers remain in achieving effective luminalization of TNBC. Further investigation is warranted.

This study relied on only two cell lines, MCF7 (luminal) and MB231 (TNBC, basal-like), and did not evaluate the effects of LCFAs on other breast cancer subtypes (e.g., HER2-positive, luminal B, other TNBC lines). Without validation in in vivo systems such as mouse models or patient-derived tissues, it is unclear whether the observed molecular changes also occur in a real tumor environment. Indeed, mouse BCA models or human BCAs show pro-metastatic ability of LCFAs in cancer cells [102,103,104]. Finally, although the clinical implications of OA and PA were discussed in the context of hormone therapy and dietary patterns, such interpretations must be made with caution. Dietary OA intake or supplementation cannot yet be linked to treatment outcomes without robust clinical or epidemiological validation.

## 4. Materials and Methods

### 4.1. Cell Lines

The human BCA cell lines MCF7 (CVCL_0031) and MB231 (CVCL_0062) were purchased from Dainihon Pharmaceutical Co. (Tokyo, Japan). Cells were cultured in phenol red-free Dulbecco’s Modified Eagle Medium (DMEM) supplemented with 10% charcoal-stripped fetal bovine serum (FBS; Sigma Chemical Co., St. Louis, MO, USA) at 37 °C in a humidified atmosphere containing 5% CO_2_. LA, EA, OA, PA, and LNA (Wako Pure Chemical Corporation, Osaka, Japan) were used at a concentration of 50 μM for 48 h. LCFAs were dissolved in 70% ethanol and then added to the culture medium to reach the desired concentration. 17β-estradiol (E2; 10 μM) and TAM (1 μM), also purchased from Wako Pure Chemical Corporation, were used for 48 h treatments.

### 4.2. MTS [3-(4,5-Dimethylthiazol-2-yl)-5-(3-Carboxymethoxyphenyl)-2-(4-Sulfophenyl)-2H-Tetrazolium] Assay

MTS assays were performed using the CellTiter 96 aqueous one-solution cell proliferation assay kit (Promega Biosciences Inc., San Luis, CA, USA). The plates were read using a Multiskan FC (Thermo Fisher Scientific, Waltham, MA, USA) microplate photometer at a wavelength of 490 nm. The MTS value of cells cultured with the control oligonucleotide was used as the control.

### 4.3. Mitochondrial Imaging

Mitochondrial function was assessed using fluorescent probes. After treatment with or without LCFAs (50 μM), cells were incubated with the respective probes for 30 min at 37 °C, then imaged using an all-in-one fluorescence microscope (BZ-X710 microscope, Keyence, Osaka, Japan). A total of 1000 cells were observed using a 40× objective with 20 fields of view. Fluorescent intensities were represented as relative values. We used DHR123 (100 μM, Dojindo, Kumamoto, Japan) to measure mitochondrial H_2_O_2_, MitoSOX (mitochondrial superoxide) (10 μM, AAT Bioquest Inc., Sunnyvale, CA, USA) to assess oxidative stress, mitoGreen (100 nM, PromoCell GmbH, Heidelberg, Germany) to assess mitochondrial volume, and TMRE (200 nM, Sigma-Aldrich, St. Louis, MO, USA) to assess mitochondrial membrane potential in accordance with the manufacturer’s instructions.

### 4.4. Reverse Transcription Polymerase Chain Reaction (RT-PCR)

To assess human and murine mRNA expression, RT-PCR was performed using 0.5 μg of total RNA extracted from the three cell lines with the RNeasy Kit (Qiagen, Germantown, MD, USA). Primer sets (listed in Table 2) were synthesized by Sigma Genosys (Ishikari, Japan). PCR products were separated on a 2% agarose gel and stained with ethidium bromide. β-actin mRNA was amplified as an internal control. The signals with ethidium bromide staining were quantified using the NIH ImageJ software (version 1.52, Bethesda, MD, USA). Signals are normalized to ACTB expression and expressed as 100% relative to the MCF7 control.

### 4.5. Sphere Assay

Cells (1000 cells/well) were seeded into uncoated bacteriological 35 mm dishes (Corning Inc., Corning, NY, USA) using 3D Tumorsphere Medium XF (Sigma-Aldrich). Cells were cultured with or without LCFAs (50 μM). After 7 d, sphere images were captured digitally, and sphere numbers were quantified using the NIH ImageJ software (version 1.52, Bethesda, MD, USA). The number of spheres was counted manually, with a size cutoff of 50 μm.

### 4.6. Protein Extraction

Protein was extracted from BCA cells following our previously reported protocol [105]. Whole-cell lysates were prepared using RIPA buffer supplemented with 0.1% sodium dodecyl sulfate (SDS) (Thermo Fisher Scientific, Tokyo, Japan), as described previously. Subcellular fractions were obtained using the Cell Fractionation Kit (Abcam, Cambridge, UK) according to the manufacturer’s instructions. Protein concentrations were measured using the Protein Assay Rapid Kit (Wako, Osaka, Japan).

### 4.7. Immunoblot Analysis

Cell lysates were separated by 10% SDS–polyacrylamide gel electrophoresis and transferred onto nitrocellulose membranes. Membranes were incubated with the anti-ERα primary antibody, followed by the peroxidase-conjugated IgG secondary antibody (P0217; Dako, Glostrup Municipality, Denmark). Immune complexes were visualized using the Fusion Solo S imaging system (M&S Instruments Inc., Osaka, Japan). Band images were captured digitally, and band intensities were quantified using the NIH ImageJ software (version 1.52, Bethesda, MD, USA).

### 4.8. Immunocytochemistry

BCA cells were cultured on chamber slides with or without OA (50 µM) or PA (50 µM) for 48 h. Cells were fixed with 4% paraformaldehyde in phosphate-buffered saline (PBS) for 15 min at room temperature and washed with PBS. Permeabilization was performed using 0.2% Triton X-100 in PBS for 10 min at room temperature. Cells were then incubated with the anti-ERα antibody (SP1, Alexa Fluor 555-labeled, 1:100 dilution, ab282199, Abcam) for 4 h at room temperature, followed by DAPI staining (1 μg/mL; Dojindo, Kumamoto, Japan) for 5 min. Fluorescence imaging was performed using a BZ-X710 microscope (Keyence, Osaka, Japan). For ER expression, 1000 cells were observed using a 40×objective with 20 fields of view. The fluorescence intensity is shown relatively, and the positive rate was not defined.

### 4.9. Mitochondrial Stress Test (Seahorse Assay)

Mitochondrial respiration and ATP production were analyzed using the Seahorse XF Analyzer (Agilent Technologies, Santa Clara, CA, USA) to measure the extracellular flux in live cells. Following 48 h of LCFA treatment (50 μM), cells were seeded into XF plates at a density of 2 × 10^4^ cells/well and incubated overnight. One hour before the assay, the medium in the XF plate was replaced with Seahorse XF DMEM (Agilent Technologies, Santa Clara, CA, USA). The Mito Stress Test (Seahorse XF Cell Mito Stress Test Kit; Agilent Technologies) was performed per the manufacturer’s instructions. The OCR was measured following sequential injections of 2 µM oligomycin, 0.5 µM carbonyl cyanide-p-trifluoromethoxyphenylhydrazone (FCCP), and 0.5 µM rotenone/antimycin A. OCR values were normalized to the total cell number.

### 4.10. Glycolytic Stress Test

The ECAR of BCA cells was measured using a modified glycolytic stress test with the Seahorse XFe24 Extracellular Flux Analyzer and XF24 FluxPaks (Agilent Technologies). Then, BCA cells were treated with LCFAs (50 μM) 48 h in 6-well plates prior to Seahorse analysis. Cells (1 × 10^4^ cells/well) were then seeded in the XF Base Medium (Agilent Technologies) supplemented with 200 mM L-glutamine and 5 mM HEPES, as recommended by the manufacturer for glycolytic assays. The sensor cartridge was rehydrated the day before the assay by adding 1 mL of the XF Calibrant to each well and incubating at 37 °C until use. The cartridge injection ports were loaded with the following compounds in sequence to achieve the indicated final concentrations: 10 mM glucose, 1 µM oligomycin, a combined injection of 1 µM rotenone, 5 µM antimycin A, and finally 50 mM 2-deoxyglucose. The combined rotenone/antimycin treatment was used to assess the contribution of mitochondrial respiration to ECAR by accounting for respiratory acidification resulting from glycolytic pyruvate entering the TCA cycle.

### 4.11. Detection of microRNA

Total RNA was extracted using the miRNeasy Kit (Qiagen, Hilden, Germany). Individual microRNAs were quantified using TaqMan MicroRNA Assay Kits, with U6 snRNA used as the internal control.

### 4.12. Inhibition of microRNA

MicroRNA inhibitors (S-TuD) for miR-34a, miR-21, and miR221 were purchased from WAKO. Cells were treated with each inhibitor at 2 μM for 24 h.

### 4.13. Statistical Analysis

Statistical significance was determined using analysis of variance (ANOVA) with Bonferroni correction, performed using the InStat software (version 3.0; GraphPad Software, Inc., La Jolla, CA, USA). A *p*-value of < 0.05 (two-sided) was considered statistically significant.

## 5. Conclusions

**Figure 7:**
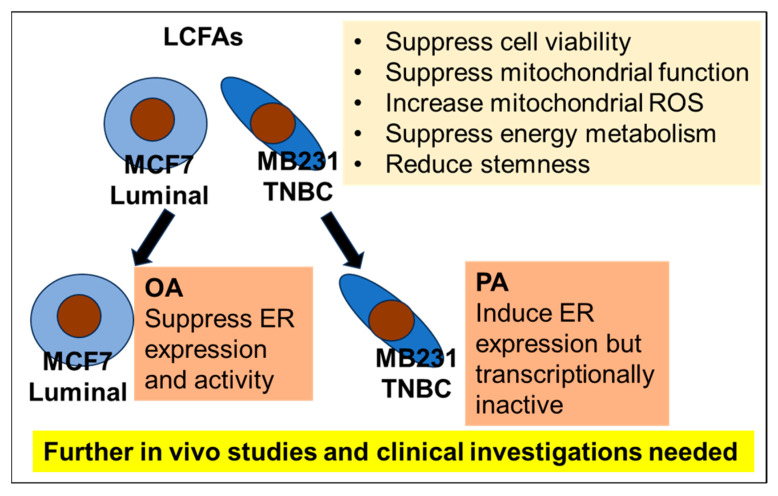
LCFAs modulate mitochondrial function, energy metabolism, and cellular stemness in breast cancer cells. Notably, OA suppresses ER expression and activity in luminal-type MCF7 cells. In contrast, PA induces ER re-expression in TNBC MB231 cells; however, the receptor remains transcriptionally inactive. Further in vivo studies and clinical validation are necessary to determine the translational relevance of these findings. LCFA, long-chain fatty acids; ROS, reactive oxygen species; TNBC, triple-negative breast cancer; MB231, MDA-MB231; OA, oleic acid; ER, estrogen receptor; PA, palmitic acid.

This study demonstrates that long-chain fatty acids (LCFAs) modulate key cellular processes—including mitochondrial function, stemness, and energy metabolism—in breast cancer (BCA) cell lines (Figure 7). Notably, oleic acid (OA) reduced ERα expression and activity in luminal-type MCF-7 cells, suggesting a possible mechanism by which fatty acids could influence hormone responsiveness. Conversely, palmitic acid (PA) upregulated ERα expression in MDA-MB-231 cells, although the receptor remained transcriptionally inactive. While these findings offer novel insights into how fatty acid metabolism may affect hormonal signaling pathways in breast cancer cells, they are based solely on in vitro experiments using two cell lines. Further in vivo studies and clinical investigations will be required to determine the relevance of these mechanisms to patient care and dietary recommendations.

## Figures and Tables

**Table 1:** Effect of LCFAs on MCF7 and MB231 cells.

LCFA	IC50 (μM)		MMP (%) *		MtVol (%) *		MtROS (%) *		Basal OCR (%) *	
	MCF7	MB231	MCF7	MB231	MCF7	MB231	MCF7	MB231	MCF7	MB231
LA	78	129	77	42	65	21	175	140	NT	NT
EA	198	NA	76	40	37	20	156	50	NT	NT
OA	192	198	108	60	52	22	130	83	35	67
PA	224	58	82	102	17	34	138.5	105	30	61
LNA	138	176	92	80	76	140	137.5	95	NT	NT

* Each value was expressed as a percentage of the control. The standard deviation of each value is under 10%. LCFA, long-chain fatty acid; LA, linoleic acid; EA, elaidic acid; OA, oleic acid; PA, palmitoic acid; LNA, α-linolenic acid; IC, inhibitory concentration of viability; MMP, mitochondrial membrane potential; MtVol, mitochondrial volume; MtROS, mitochondrial reactive oxygen; it is the mean value of H_2_O_2_ and superoxide; basal OCR, basal oxygen consumption rate; NA, not applicable; EA did not reach IC50; NT, not tested.

**Table 2:** Polymerase chain reaction (PCR) primers and antibodies.

RT-PCR Primers			
Gene	ID	Upper (5′ to 3′)	Lower (5′ to 3′)
*ACTB*	NM_001101.3	ggacttcgagcaagagatgg	agcactgtgttggcgtacag
*CD24*	BC064619.1	atgggcagagcaatggtgg	ccacgaagagactggctgtt
*OCT3*	BC117437.1	gaaggatgtggtccgagtgt	gtgaagtgagggctcccata
*CDH1*	BC146662.1	cgtcctgggcagagtgaatt	gctctgtcaccttcagccat
*CK18*	NM_000224.3	ccgcatcgttctgcagattg	tctgactcaaggtgcagcag
*c-Myc*	NM_002467.4	ttcgggtagtggaaaaccag	cagcagctcgaatttcttcc
*PGC-1α*	BC156323.1	gtgaagaccagcctctttgc	aatccgtcttcatccacagg
*ESR1*	JF810888.1	gctccgcaaatgctacgaag	agatctccaccatgccctct
Antibodies			
Target	RRID	Company	
ERα	AB_2617128	Cell Signaling Technology, Danvers, MA, USA	
GAPDH	AB_2107448	Abcam, Waltham, MA, USA	
TBP	− *	Abcam, Waltham, MA, USA	

* Not registered. The catalog number is ab300656. ACTB, beta-actin; Oct3, octamer-binding transcription factor 3; CDH1; E-cadherin; CK, cytokeratin; PGC1α, peroxisome proliferator-activated receptor gamma coactivator 1-α; ESR1, αER; GAPDH, glyceraldehyde-3-phosphate dehydrogenase; TBP, TATA-binding protein; RT-PCR, reverse transcription polymerase chain reaction; RRID, Research Resource Identifier.

## Data Availability

Data is contained within the article.

## Abbreviation

BCA, breast cancer; ER, estrogen receptor; HER, human epidermal growth factor receptor; ERE, estrogen response element; TNBC, triple-negative breast cancer; GATA3, GATA-binding protein 3; FOXA1, forkhead box protein A1; miRNA, microRNA; LCFA, long-chain fatty acid; PA, palmitic acid; OA, oleic acid; LA, linoleic acid; EA, elaidic acid; LNA, α-linolenic acid; PI3K, phosphatidylinositol 3-kinase; MMP, mitochondrial membrane potential; Oct3, octamer-binding transcription factor 3; PGC1α, peroxisome proliferator-activated receptor gamma coactivator-1α; OCR, oxygen consumption rate; ECAR, extracellular acidification rate; E2, estradiol; TAM, tamoxifen; PgR, progesterone receptor; TFF1, trefoil factor 1; BCXL2, B-cell/CLL lymphoma 2; ROS, reactive oxygen species; EMT, epithelial–mesenchymal transition; PPAR, peroxisome proliferator-activated receptor; SRC, steroid receptor coactivator; CRB, cAMP response element-binding protein; CBP, CREB-binding protein.
